# Surface tension and viscosity of liquid Pd_43_Cu_27_Ni_10_P_20_ measured in a levitation device under microgravity

**DOI:** 10.1038/s41526-019-0065-4

**Published:** 2019-02-25

**Authors:** Markus Mohr, Rainer K. Wunderlich, Kai Zweiacker, Silke Prades-Rödel, Romuald Sauget, Andreas Blatter, Roland Logé, Alex Dommann, Antonia Neels, William L. Johnson, Hans-Jörg Fecht

**Affiliations:** 1 Institute of Functional Nanosystems FNS, Ulm University, Albert-Einstein-Allee 47, 89081 Ulm, Germany; 2 Center for X-ray Analytics, Empa Swiss Federal Laboratories for Materials Science and Technology, Überlandstrasse 129, CH-8600 Dübendorf, Switzerland; 3 PX Services SA, Boulevard des Eplatures 42, 2304 La Chaux-De-Fonds, Switzerland; 4 Thermomechanical Metallurgy Laboratory – PX Group Chair, Ecole Polytechnique Fédérale de Lausanne (EPFL), Neuchâtel, Switzerland; 5 California Institute of Technology, 1200 East California Boulevard, Pasadena, CA USA

## Abstract

Here we present measurements of surface tension and viscosity of the bulk glass-forming alloy Pd_43_Cu_27_Ni_10_P_20_ performed during containerless processing under reduced gravity. We applied the oscillating drop method in an electromagnetic levitation facility on board of parabolic flights. The measured viscosity exhibits a pronounced temperature dependence following an Arrhenius law over a temperature range from 1100 K to 1450 K. Together with literature values of viscosity at lower temperatures, the viscosity of Pd_43_Cu_27_Ni_10_P_20_ can be well described by a free volume model. X-ray diffraction analysis on the material retrieved after the parabolic flights confirm the glassy nature after vitrification of the bulk samples and thus the absence of crystallization during processing over a wide temperature range.

## Introduction

Bulk metallic glasses (BMGs) represent a new development in materials science with the major advantage to possess superior mechanical properties (and others) compared with materials in their conventional crystalline state. Metallic glasses, solid metallic materials with a disordered liquid-like atomic-scale structure, are formed when they get cooled faster from the liquid state than a critical cooling rate. The higher the glass-forming ability (GFA) of a metallic glass, the lower is the critical cooling rate and the better it is suitable for industrial applications. Some of the most robust BMG alloys in terms of critical cooling rates and oxidation/corrosion resistance are based on precious metal–metalloid alloy systems, such as Pd-, Pt-, and Au-based alloys, combined with typically 20 at% of phosphorous. Within this material class, the metallic glasses composed of Pd-Cu-Ni-P and Pd-Ni-P have an outstanding GFA, reflected by their very high reduced glass temperature.^1–5^ During cooling of a liquid, the increasing thermodynamic driving force for crystallization and the reducing atomic kinetics are competing.^6^ The formation of a glass during cooling of a liquid demands the bypass of the nose of the so-called temperature-time transformation (TTT) diagram, describing the time in isothermal conditions, after which considerable crystal nucleation occurs.^7^ The strong decrease in atomic kinetics during cooling is one important factor in order to obtain BMGs during industrial processing, such as casting or injection molding. The strong increase of viscosity during cool down, and the demand to achieve vitrification of the liquid also establishes boundary conditions for the right choice of process parameters for industrial production procedures. Also, for superplastic forming technologies, precise knowledge of the temperature-dependent viscosity of the alloy is of importance.

Thus, it is important to provide basic thermophysical property data over a wide temperature range to design production processes and models for supporting process simulations.

The precise measurement of thermophysical properties such as surface tension and viscosity of metallic alloys in their liquid phase (at high temperatures) demand clean conditions, especially the absence of foreign materials that could contaminate the surface or bulk of the measured liquid sample. This makes processing under ultra-high vacuum or inert gas mandatory. Additionally, the high reactivity of typical metallic melts makes containerless methods necessary for many metallic alloys. A very versatile containerless processing method that offers wide applicability to electrically conductive samples is electromagnetic levitation (EML).^8–12^ This method enables the determination of surface tension and viscosity by the oscillating drop method,^13^ where surface oscillations of the liquid sample are excited, observed, and analyzed.

However, under earth’s gravitational conditions, a liquid sample in its natural geometry or levitated by an electromagnetic positioning field will be considerably deformed. For levitated drops, this leads to a split of oscillating frequencies.^13,14^ In addition, simultaneous temperature and levitation control is limited under normal 1*g* gravitational conditions, since the positioning field required for lifting the samples may already heat the sample to significant temperatures, even beyond the melting point (especially true for low melting BMGs). Furthermore, the fluid flow in the constantly heated, deformed droplet under terrestrial conditions is not well controlled (laminar to turbulent transition), which makes it necessary to perform the experiments in reduced gravity conditions (microgravity, µ*g*).

One possibility to reach µ*g* for a short period of time (10–20 s) are parabolic flights, such as those performed by Novespace using an Airbus A310.

The presented results were collected during two parabolic flight campaigns in year 2016 and 2017, employing the EML facility TEMPUS (’Tiegelfreies elektromagnetisches Prozessieren unter Schwerelosigkeit’, engl. ‘Containerless electromagnetic processing under weightlessness’).^15^ We present the surface tension and viscosity of Pd_43_Cu_27_Ni_10_P_20_ in the liquid phase and show that the temperature dependence of the viscosity of Pd_43_Cu_27_Ni_10_P_20_ can be well described by a free volume model. Furthermore, we confirm the absence of long-range order, characteristic for BMGs, using X-ray diffraction (XRD). The samples morphology is analyzed by X-ray computed tomography (CT) before and after the flights.

## Results

**Fig. 1:**
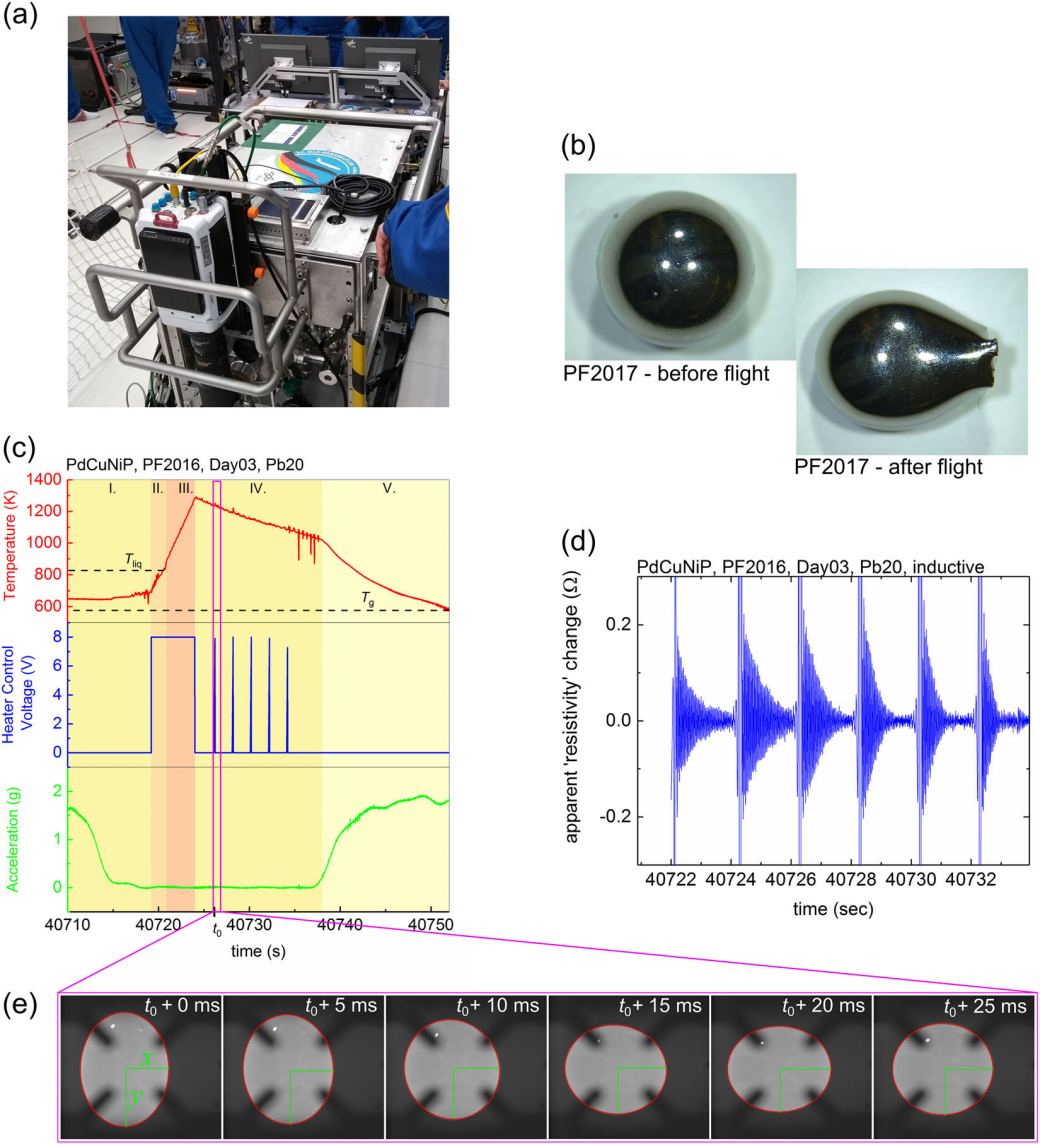
**a** Photograph of the TEMPUS facility on board the parabolic flight airplane. **b** Photographs of the samples before and after the processing in the parabolic flight 2017 are shown. The deformation of the sample after the flight is due to the contact of the sample with the sample holder at the end of the parabola. **c** Temperature-time profile of processing in the electromagnetic levitator on board a parabolic flight (red). The control voltage of the rf-heater (blue) shows pulses for the excitation of surface oscillations. The level of vertical acceleration (green) shows the ~20-s time window of µ*g*. **d** Variation of the high-pass filtered “apparent” electrical resistivity as a function of time—the exponentially decaying surface oscillations can be detected after heater turn-off and after every heater pulse. **e** Series of frames recorded by the high-speed camera, showing the surface oscillations of the droplet

The photograph in Fig. 1a gives an impression of the TEMPUS facility on board the parabolic flight airplane. The samples are contained in a sample chamber during flight, and the desired sample is brought to the experiment chamber for processing in several subsequent parabolas. A coil system connected to two rf-generators is used to position and heat the sample independently. The sample temperature is measured using a pyrometer, while two high-speed cameras observe the sample during processing. Typical sample diameters are between 6 and 7 mm. Figure 1b shows photographs of the PdCuNiP sample before and after the parabolic flight in 2017.

In Fig. 1c, a representative temperature-time profile in the µ*g* phase during processing of Pd_43_Cu_27_Ni_10_P_20_ is shown. The sample temperature is shown in red in the first diagram, while the control voltage of the rf-heater with pulses for the excitation of surface oscillations is shown in the second diagram in blue. Additionally, the level of vertical acceleration is shown in green below. In every parabola, after positioning the sample (see I in Fig. 1c), the heater is turned on to melt the sample (see II in Fig. 1c) and overheat the liquid melt by about 450 K (see III in Fig. 1c). Afterwards, the heater is turned off (IV in Fig. 1c), giving the sample the opportunity to cool down by heat radiation and heat conduction in the inert gas atmosphere until the end of the µ*g* phase (see IV and V in Fig. 1c).

A series of short heat pulses is applied in order to initiate surface oscillations of the liquid droplet. The excitation of surface oscillations is detected by two different methods.

The surface oscillations, effectively being a modulation of the sample diameter, also modulate the apparent impedance of the heater coil circuitry. This way, the sample oscillations are detected inductively by an electronic measurement equipment, the so-called sample coupling electronics (SCEs). Figure 1d shows the high-pass filtered apparent electrical sample resistivity determined from the modulated impedance of the heater circuit. As seen in Fig. 1d, after turning off the heater after the initial melting step, the sample radius oscillates with an exponentially decaying amplitude. The time constant varies as a function of temperature and is related to the viscosity, as described below in more detail. The same happens also after every heater pulse.

The second approach to detect the surface oscillations utilizes high-speed videos (at typical frame rates between 150 and 200 Hz) that are taken from the sample during the cooling period. Figure 1e shows six frames recorded after the time *t*_0_, when the heater pulse was turned off. The initial deformation is shown in the frame taken at *t*_0_, and evidently the sample starts to oscillate thereafter. An edge detection algorithm is used to obtain several deformation measures, such as the *X* and *Y* radius of the sample, shown in Fig. 1e, from which the oscillation amplitudes can be derived.

In all parabolas, at the end of the µ*g* phase, the sample touched the sample holder pedestal while still in the liquid phase. This also gives rise to the non-spherical shape of the samples observed after processing in 2017 (see Fig. 1b) and in 2016 (see Fig. 4). No sign of solidification such as recalescence was observed during levitation in the µ*g* phase before. During all processing cycles, no sign of surface precipitates could be observed on either sample. No visible signs of surface contamination could be observed when the sample was taken out of the TEMPUS facility after processing.

### Surface tension and viscosity

**Fig. 2:**
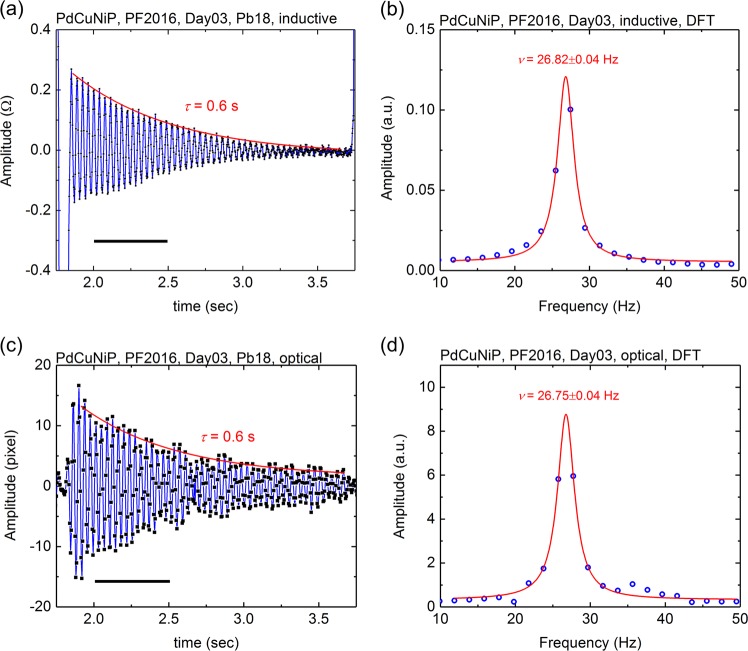
**a** Oscillation amplitude, as detected by the inductive method as a function of time. **b** Discrete Fourier transformation (DFT) spectrum of the amplitude variation between 2.0 and 2.5 s. **c** Amplitude of the optically determined surface oscillations. **d** DFT spectrum of a selected time slice

The surface tension *σ* can be deduced from the samples surface oscillation frequency *ν*_R_. In order to obtain the surface oscillation frequency from the optically and inductively measured amplitudes (see Figs. 2a, c), Fourier spectra are calculated through a dedicated discrete Fourier transformation (DFT) algorithm. These spectra are used to obtain the surface oscillation frequency at different temperatures, by fitting a Lorentzian function (see Figs. 2b, d for Fourier spectra of the optically and inductively obtained signals). The surface tension *σ*, the sample mass *M*, and the oscillation frequency *ν*_R_ are generally related by^16^

1 $$\sigma = \frac{3}{8}\pi \,{\kern 1pt} \nu _R^2M$$

and hence, the oscillation frequency at different temperatures during the sample cooling period can be determined (see IV in Fig. 1c).

Due to internal friction in the liquid state, the surface oscillations are considerably damped, exhibiting an exponentially decreasing oscillation amplitude, according to *A*(*t*)=A_0_exp(*-t/τ*). While Fig. 1d shows a general overview of six electromagnetic pulses in a time window of ca. 10 s., Figs. 2a, c show details of damped oscillations after one single heater pulse. The damping time constant *τ* of the surface oscillations can be obtained by proper fitting of the signal envelope. The viscosity of the liquid can be obtained by^17^

2 $$\eta = \frac{3}{{20\pi }}\frac{M}{R}\frac{1}{\tau }$$

where *R* is the averaged radius of the sample. See Methods section for further details.

The inductive method is less prone, but not immune to effects of sample translation, rotation, and precession. As a consequence, most of the sample oscillation analysis was performed with the inductive method.

**Fig. 3:**
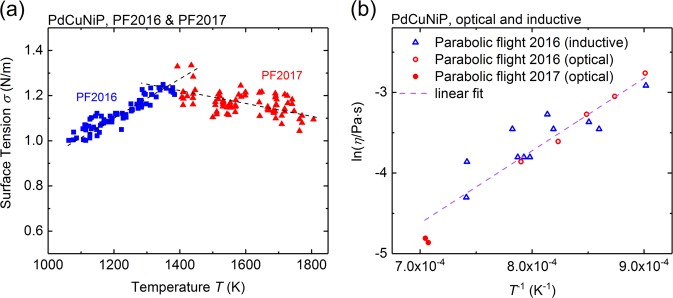
**a** Surface tension data obtained in both parabolic flight campaigns 2016 and 2017. **b** Arrhenius plot of the viscosity obtained for liquid Pd_43_Cu_27_Ni_10_P_20_ in the temperature range between 1100 K and 1450 K

In Fig. 3a, the surface tension as a function of temperature is shown as determined in the parabolic flight in 2016 and 2017. The first campaign covered a temperature range of 1050 K – 1400 K, whereas in the second one, a temperature range of 1350 K–1850 K was investigated.

The surface tension values in the lower temperature range show a positive temperature coefficient. Reduction of surface tension is often observed by surface-active species, while, less of them would be adsorbed on the surface at higher temperatures. The same effect could be due to a temperature-dependent surface segregation of surface-active elements of the alloy, such as phosphorus. The values of the higher temperature range can be well represented by a linear temperature dependence having a negative temperature coefficient according to:

3 $$\sigma \left( T \right) = \left( {1.53 \pm 0.09} \right)-\left( {4.28 \pm 0.67} \right) \times 10^{ - 4} \times \left( {T-827\,\mathrm{K}} \right)\,{\mathrm{N/m}}$$

The formula represents an average of the data obtained from four parabolas. The extrapolation of the obtained surface tension data to the liquidus temperature gives *σ*(*T*_liq_) = (1.53 ± 0.10) N/m.

In Fig. 3b, the viscosity of Pd_43_Cu_27_Ni_10_P_20_ is shown in an Arrhenius plot. The values comprise data obtained from the inductive and optical method in the parabolic flight campaign 2016. The scatter of the viscosity values shown in Fig. 3b can partly be understood by the quality of the measured data. Sample movement and the related distortion of the sample edges contribute to disturbances of the optical data. Also, slow modulations of the apparent electrical conductivity or the sample *X* or *Y* radius can be present due to the precession of the sample or possibly due to mode jumps between the degenerated *Y*_2,m_ modes. These phenomena are limiting the precision of viscosity determination.

At the high temperatures (1100 K – 1450 K) far above the glass transition temperature, the data can be satisfactorily represented by an Arrhenius dependence: *η*(*T*) = *η*_0_ × exp(*E*_A_/*k*_B_*T*) with *η*_0_ = (18.2 ± 9) µPa s and *E*_A_ = (0.77 ± 0.07) eV. This is evident from the good linear fit shown in Fig. 5.

In the higher temperature range, investigated in parabolic flight campaign 2017, only one data point at 1420 K could be analyzed, for the higher temperatures, the sample oscillation was not taking place in a single mode or the sample was performing movements, which obscured the exponential damping of the surface oscillations.

### Structural and chemical analysis

Samples were weighted before and after they were processed in the TEMPUS sample chamber. The mass was determined as 1.3387 g (precision ±0.0004 g) and afterwards as 1.3385 g. Therefore, the mass loss can be estimated to be lower than the precision of the balance (~ 0.4 mg). The sample was heated six times to 1100– 1200 °C. The time for which the temperature was above 900 °C was about 7 s in each parabola. The evaporation rate is therefore below 10 µg/s in the temperature range between 900 °C and 1200 °C.

The energy dispersive X-ray spectroscopy (EDX) analysis of as-cast and processed samples shows homogeneous mixture of all constituents of the alloy. Within the measurement uncertainty of the EDX analysis, no change of sample composition was measurable, which is expected, considering the very low mass loss during processing.

Furthermore, the samples returned from the parabolic flights were analyzed using XRD and CT methods in order to elaborate the homogeneity of the solidified microstructure and to determine the presence or absence of voids.

**Fig. 4:**
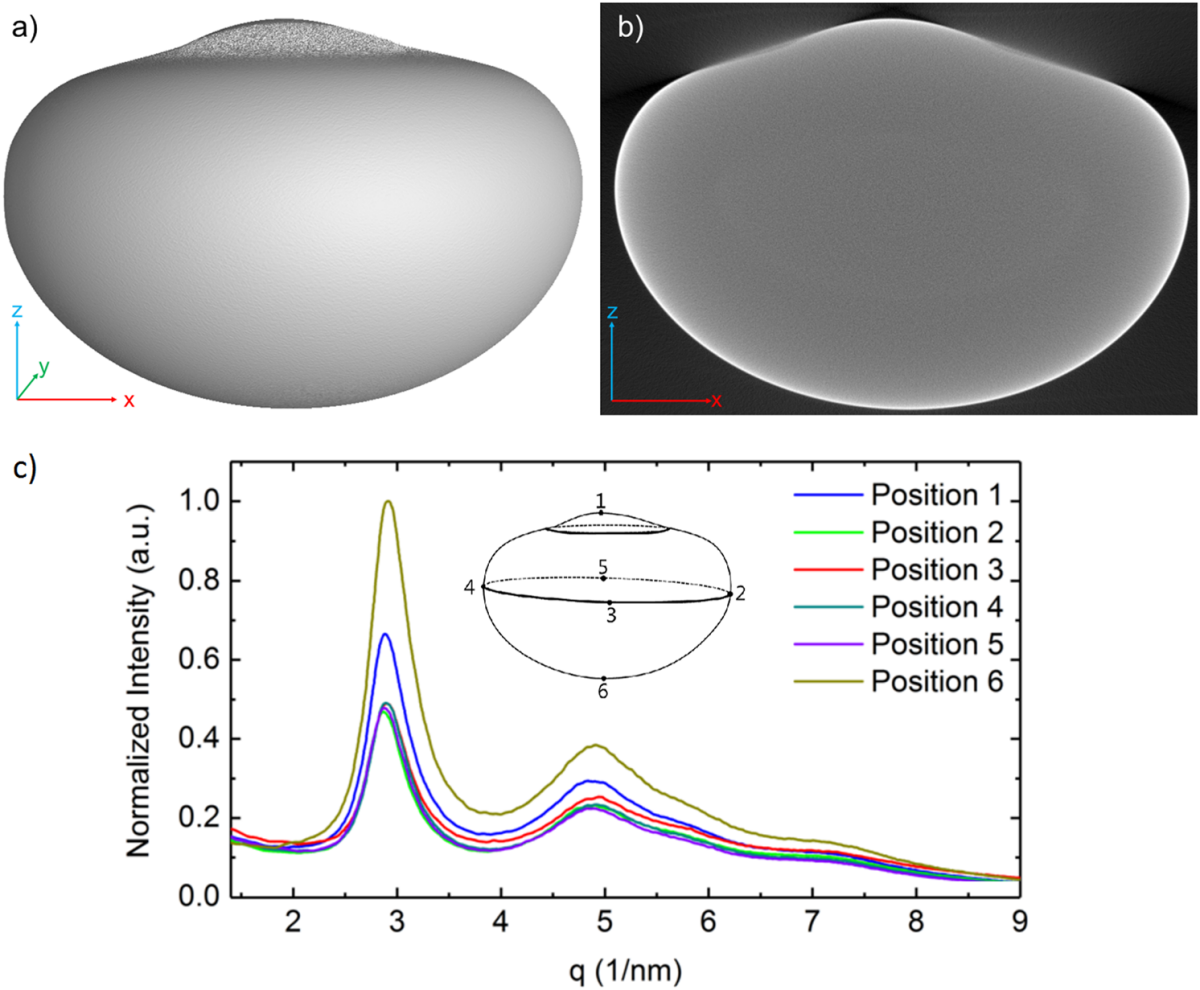
**a** Three-dimensional (3D) reconstructed volume of a X-ray computed tomography (CT) scan; **b** an example slice of Pd_43_Cu_27_Ni_10_P_20_. **c** Diffraction patterns obtained after the PF experiment (PF 2016) the inset shows a sketch of the vitrified sample shape

During the processing cycles in the TEMPUS, the sample was repeatedly molten and vitrified. By the forced gas cooling with He–Ar gas mixture and the final contact of the sample with the sample holder at the end of the µ*g* cycle, the cooling rate was higher than the critical cooling rate of Pd_43_Cu_27_Ni_10_P_20_, thus avoiding crystallization. Typically, the phase change from liquid to solid is concurrent to a reduction in volume. This volume reduction can cause small pores and voids within the sample. Figure 6 shows an absorption X-ray CT image and the volumetric reconstruction of the melted and subsequent solidified Pd-alloy sample. Absorption-based X-ray CT is a powerful nondestructive technique to visualize the interior features within solid objects and obtaining digital information on three-dimensional (3D) geometries (Fig. 4a). Here, CT is utilized to probe the solidified Pd-alloy specimen to determine larger flaws. An absorption X-ray CT image, typically referred to as slice corresponds to a certain thickness of the sample, each pixel of a slice is corresponding to a volume element (voxel). Figure 4a shows the result of complete volumetric reconstruction after six melt-solidification cycles. The reconstruction is composed of 1392 slices and each slice can be analyzed separately. The gray levels in the displayed representative slice, i.e., Fig. 4b, correspond to the X-ray attenuation, which reflects the proportion of X-rays scattered or absorbed as they pass through each voxel. X-ray attenuation is mainly a function of the X-ray energy, the density and composition of the material being imaged. The slice shown in Fig. 4b shows the absence of any kind of internal features. However, a strong beam-hardening effect on the outer periphery of the sample slice (bright contrast) is visible due to nonlinearity of the absorption coefficient of the non-monochromatic X-ray beam; this effect can safely be neglected. The sample, being spherical during the microgravity phase, is vitrified in the shape shown in Figs. 4a, b due to the deformation happening after the contact with the sample pedestal at the end of the microgravity phase.

XRD on the other hand is based on constructive interference of monochromatic X-rays on periodic arrangements of atoms or molecules in a crystalline sample. Amorphous materials do not possess that periodicity at a long range. Here, the scattering of X-rays by atoms is considered; hence strong and narrow reflections that represent long-range ordering are absent and instead broad diffuse peaks will be present that are indicative of nearest neighbor distances.

Six locations for XRD measurements were chosen based on the sample reconstruction from CT. The sample shows a clear rotational symmetry along the axis drawn between points 1 and 6, points 2–4 are located around the largest circumference of the displayed reconstructed volume. Figure 4c shows six individual measurements from position 1 to 6 around the surface of the Pd-alloy sample from the first parabolic flight experiment. All of the resulting diffraction patterns show symmetrical broad first peaks with maxima at 2.88 Å and second peaks maxima at 5.022 Å, the symmetricity of the first peak and the ratio between first and second peak maxima (1.74) are representative of amorphous metallic samples.^18,19^ The diffraction patterns for the experiments performed in the parabolic flight campaign 2017 are comparable, hence not shown here.

## Discussion

During two parabolic flight campaigns, the surface tension of Pd_43_Cu_27_Ni_10_P_20_ was successfully measured in the temperature range of 1050 K–1850 K. While in 2016, at the lower temperature range (below 1300 K), the surface tension was probably influenced by surface-active species leading to a positive temperature coefficient, in the higher temperature range, a negative temperature coefficient is obvious. Even though we could not find measurable composition gradients to the sample surfaces by EDX measurements, surface segregation of a small fraction of surface-active species, could be responsible for a change of the surface tension. Further investigations on surface segregation and influences of adsorbents from the gas atmosphere are necessary to explain this phenomenon.

In this work, the viscosity of Pd_43_Cu_27_Ni_10_P_20_ was measured in the liquid phase in a temperature range of 1100 K – 1450 K, above the liquidus temperature.

Directly measured values for the viscosity of liquid Pd_43_Cu_27_Ni_10_P_20_ are scarce in literature, however, measurements of the viscosity at and above the glass temperature are available. The only direct measurement of viscosity of liquid PdCuNiP (Pd_40_Cu_30_Ni_10_P_20_) was performed by Haumesser et al.^20^ using a gas-film levitation technique. The investigated temperature range was 880 K–1137 K, where the viscosity varied between 69 mPa s and 11 mPa s. In contrast to that, our measurements were performed in a temperature range from 1110 K to 1420 K and we obtained viscosities between 63 mPa s and 7.5 mPa s for a PdCuNiP alloy with a slightly higher Pd/Cu ratio of 43/27. The Pd/Cu ratio was shown to be a critical parameter determining the thermophysical properties in the Pd_40+x_Cu_30-x_Ni_10_P_20_ system.^4^ As was shown by Lu et al., the glass transition temperature (+7 K) and the onset of crystallization (+36 K) is higher, when the Pd/Cu ratio is increased from 40/30 (*x* = 0) to 43/27 (*x* = 3).^4^ This may also explain the slightly different viscosities obtained for slightly different PdCuNiP compositions in literature. Kato et al.^21^ investigated the deformability of Pd_42.5_Cu_30_Ni_7.5_P_20_ at and slightly above the glass transition temperature, obtaining lower viscosities than those by Fan et al., who measured a slightly different composition (Pd_43_Cu_27_Ni_10_P_20_).^22^ Measurements of Lu et al.^23^ were performed in the solid state between 610 and 680 K, and are in very good agreement with the measurements of Fan et al.^22^ performed in the same temperature range.

**Fig. 5:**
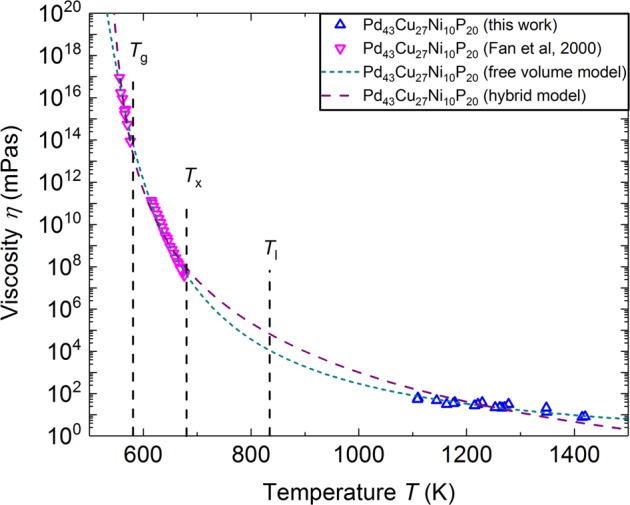
Temperature-dependent viscosity of Pd_43_Cu_27_Ni_10_P_20_, measured on board a parabolic flight (this work), and measured by parallel plate rheometry and three-point beam bending by Fan et al.^22^

The measurements done by parallel plate rheometry, dilatometry, or beam bending around and above the glass transition temperature can only cover a limited temperature range. Containerless methods can complete the available data with viscosities in the liquid phase. Figure 5 shows the viscosity of Pd_43_Cu_27_Ni_10_P_20_, obtained by Fan et al.,^22^ together with the viscosity obtained in this study. The large temperature range (~ 1000 K), covering 16 orders of magnitude in viscosity, is used to compare theoretical viscosity models.

The free volume model,^24^ expresses viscosity as

4 $$\log (\eta ) = {\kern 1pt} \frac{{A + 2B}}{{T - T_0 + \sqrt {(T - T_0)^2 + 4\nu _a\zeta _0T} }}$$

with *B* = *v*_m_*ζ*_0_log(e), where *v*_m_ is the molecular volume and *v*_a_ and *ζ*_0_ are constant parameters of the model, used in the description of the local free energy.

An alternative model is the hybrid model^25^ that divides the temperature dependence of viscosity in two temperature regimes. Decreasing temperature in the low temperature regime increases viscosity by increased chemical short-range order, while the activated annealing of flow defects at high temperatures decreases the viscosity for increasing temperature.^25^ This can be expressed as^25^

5 $$\eta (T) = \eta _0\exp \left( {\frac{E}{{k_BT}}} \right)\exp \left( {\frac{A}{{T - T_0}}} \right)$$

For the characteristic temperature, we used in both models the Kauzmann temperature *T*_K _= 507 K.^4^

Figure 5 shows the fit of both models to the experimental data. The coefficients of determination (COD), calculated for the fits show that the free volume model fits the experimental data better than the hybrid model. The determined parameters from both model fits are shown in Table 1.

**Table 1:** Parameters, determined by fitting both models to the experimental viscosity data of Pd_43_Cu_27_Ni_10_P_20_

Model	Parameters
Free volume model	*A* = –3.93, *v*_m_/*v*_a_ = 448.42, *v*_a_*ζ*_0_ = 8.91 K
Hybrid model	*η*_0_ = 2.32 × 10^–8^ Pa s, *E* = 1.32 eV, *A* = 1104 K^–1^

The possible presence of surface-active species during the parabolic flight in 2016, signified by the positive temperature dependence of the surface tension is important for the evaluation of the measured viscosity. Since an inhomogeneous coverage of the surface with adsorbents could lead to convective Marangoni flows on the surface, the measured viscosity has to be viewed with caution. Further analysis, such as magneto-hydrodynamic simulations are necessary to investigate, if inhomogeneous coverage of surface-active species would induce Marangoni convection and to what extent such Marangoni flows could affect the apparent viscosity measured by the oscillating drop method.^26^

**Fig. 6:**
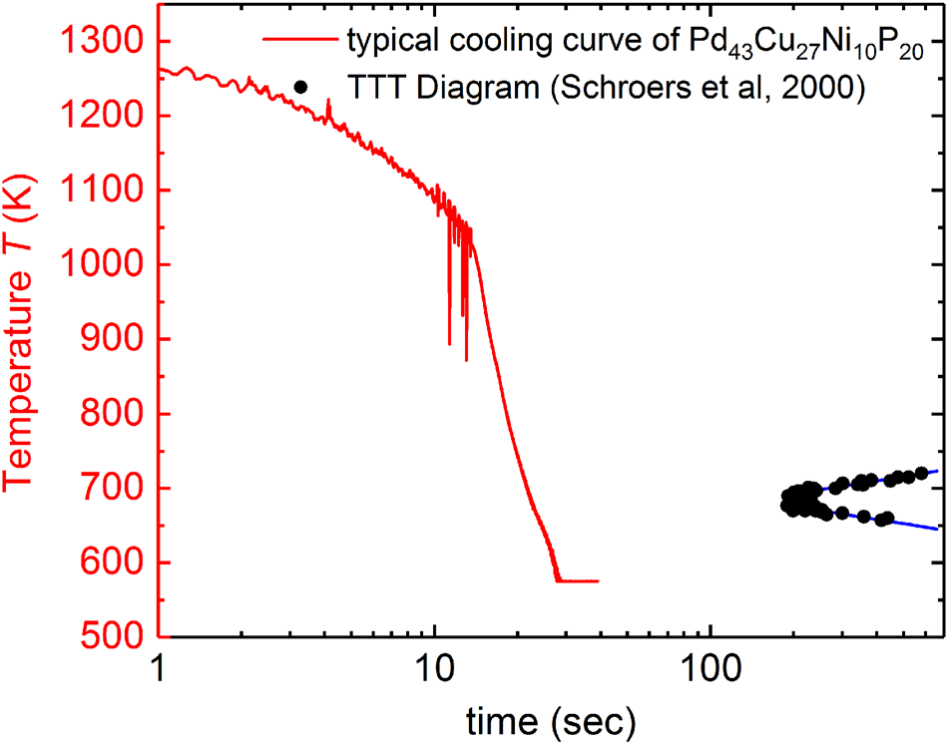
The temperature variation during the cooling is shown after the heater was turned off (red). Here, data of the last parabola performed on the sample in the parabolic flight campaign 2016 is shown. For comparison, the temperature-time transformation (TTT) diagram measured by Schroers et al.^7^ is shown

That the samples could be vitrified during the process cycles in the parabolic flight can be rationalized when the TTT diagram of Pd_43_Cu_27_Ni_10_P_20_ is compared with the cooling curve, as it is done in Fig. 6. The TTT diagram of Pd_43_Cu_27_Ni_10_P_20_ was determined by Schroers et al. and its nose was found to be at about 680 K and 200 s.^7^

The increased negative slope of the temperature, after around 15 s, is due to the contact of the sample with the sample holder at the end of the parabola. It is apparent that the sample temperature dropped fast enough to bypass the nose of the TTT diagram to prevent crystallization.

Furthermore, reflection-based XRD analyses have been performed for the both samples retrieved from the parabolic flight campaigns 2016 and 2017. The resulting diffraction patterns show the amorphous state of the specimens after the last melt and solidification cycle, it shows the symmetry of the external heat extraction geometry is maintained in the diffraction pattern, i.e., direct gas flow cools the fastest. Further, volumetric absorption-based X-ray CT showed the uniformity of the specimen, and confirmed only minimal weight loss (below 0.03%).

The surface tension and viscosity of Pd_43_Cu_27_Ni_10_P_20_ BMG alloy were measured during levitation in reduced gravity on board parabolic flights. The surface tension was measured in the temperature range from 1050 to 1850 K, while viscosity data was obtained between 1100 and 1450 K.

The viscosity evaluated in the stable liquid phase appears generally higher than the values determined by Haumesser et al.^20^ for Pd_40_Cu_30_Ni_10_P_20_. The slightly higher glass transition temperature and the larger resistance of Pd_43_Cu_27_Ni_10_P_20_ against crystallization, compared with the Cu richer Pd_40_Cu_30_Ni_10_P_20_, is in general agreement with the higher viscosity of liquid Pd_43_Cu_27_Ni_10_P_20_. A free volume model can be fit well to the temperature-dependent viscosity of Pd_43_Cu_27_Ni_10_P_20_ given in literature and the measurements in the present investigation.

Energy dispersive X-ray spectroscopy measurements have shown that samples exhibit a homogeneous chemical element distribution on the sample surface and cross section before and after processing in µ*g*.

Volumetric absorption-based X-ray CT showed the homogeneity of the specimen and the symmetry of the cooling conditions in the solidified samples. Scattering curves resulting from reflection based X-ray diffractograms show the amorphous state of the specimens after the last melt and solidification cycle. Further it was observed that the excess free volume differs, depending on the rate of heat extraction, given by the geometry of the specimen and sample pedestal.

As shown in Figs. 1c and 6, the µ*g* time was too short to cool the liquid melt below the glass transition temperature before it touched the sample pedestal. As such, it is remarkable that the SiN sample holder pedestal did not act as a heterogeneous nucleant. Studies concerned with the solidification or vitrification under µ*g* were therefore not possible in this experiments.

Considering the very small critical cooling rate of the PdCuNiP BMGs (below 0.1 K/s) and the cooling rates of ca. 20 K/s (see Fig. 1c) that are typically achievable in the TEMPUS facility, would allow the vitrification under µ*g* conditions if the µ*g* time would last longer. Vitrification on ground (at 1*g*) is accompanied by effects of the ubiquitous gravity, which can be avoided by processing under microgravity. Longer µ*g* times can be achieved by other µ*g* platforms such as the International Space Station (ISS), which also open the possibility to study further material properties, such as the specific heat capacity and thermal conductivity in the (undercooled) liquid state. Hence, preparations for the processing of noble metal-based metallic glasses, such as PdCuNiP in the EML facility (ISS-EML) on board the ISS are ongoing.

The containerless processing in TEMPUS on board parabolic flights, as presented here, is a good possibility for the measurement of thermophysical properties within µ*g* times of around 20 s, like surface tension and viscosity. Besides, it is a testing platform for sample positioning, heating, and cooling efficiency, which give important knowledge for the design of experiments with similar samples in the electromagnetic levitator ISS-EML on board the Columbus module of the ISS.

## Methods

### Sample preparation, structural, and chemical analysis

A master alloy with the composition Pd_43_Cu_27_Ni_10_P_20_ was prepared at the precious metal foundry of PX group. A one-kilo batch has been melted in a vacuum induction furnace using pure components. Purity was 99.95% for the palladium and nickel, 99.99% for the oxygen-free copper, and 99.999% for phosphorous. The obtained master alloy is then re-melted and cast in amorphous rods of 8 mm diameter in a vacuum induction furnace without any fluxing. Suitable pieces of the rod were used to prepare spheres of 6.5 mm diameter in a water-cooled copper mold using an arc melter. Their exposure to air was minimized to the time needed for sample integration in the TEMPUS facility (~20 min). The base pressure in the process chamber of the levitator was in the range of 2 × 10^-7^ mbar.

After the processing in µ*g*, the samples were analyzed by EDX, XRD, and CT. XRD was performed on a Stoe imaging plate diffractometer system (IPDS II, Stoe & Cie GmBH, Darmstadt, Germany). Two-dimensional (2D) diffraction data in reflection mode were collected at room temperature using MoKα radiation (λ = 0.71073 Å); the resulting images were then azimuthally integrated. An X-ray CT setup was utilized at 280 kV in its nominal geometry, a total of 1392 z-slices were acquired in 3 h. The CT setup was composed of a microfocus source from Finetec (model FOMR 300.03Y RT) and a flat-panel detector with 100 µm^2^ pixels from Perkin Elmer (model XRD 1611-CP3).

### Contact-less EML–TEMPUS

The experiments were performed using the electromagnetic facility TEMPUS, which has been run by DLR personnel on board a parabolic flight airplane^15^ operated by Novespace. TEMPUS consists of a process and a sample chamber, which are connected to a high vacuum pumping system and a gas circulation unit. The gas circulation system is equipped with a gas cleaning cartridge specified to impurity levels <1 ppb for O_2_ and H_2_O. The sample is heated and positioned by two different radio frequency (rf-) electromagnetic fields: a dipole field for heating and a quadrupole field for positioning. The rf-power is supplied by two rf-generators operating at frequencies of 375 kHz and 150 kHz for heating and positioning, respectively.^27^ Further details of the experimental setup and data analysis are described elsewhere.^28,29^

To determine the electrical resistivity and the sample radius of the processed liquid sample by contact-less inductive means a measurement electronics (SCEs) is attached to the facility. It measures the current, voltage, phase shift, and frequency of the rf generator that establishes the heating dipole field.^30^

Due to its high sensitivity, the SCE also allows the detection and evaluation of the surface oscillations of a liquid metallic droplet via the inductive coupling of the rf-heaters oscillating circuit and the induced current distribution in the sample. The SCE operates with a sampling rate of 400 Hz. This is very well suited for surface oscillation analysis, as their typical frequencies are in the range between 20 and 50 Hz.

The process chamber is equipped with several observation windows allowing the recording of the sample shape in two perpendicular directions using two high-speed cameras. One camera is mounted axially (for top view) along the direction of the rf-induction coil axis and the second one radially, in a direction perpendicular to the former. Both cameras are typically operating at 200 Hz, compromising between brightness and temporal resolution.

An optical pyrometer is integrated in the axial camera for temperature measurement in the range between 300 and 2100 °C. The optical pyrometer operates at a sampling rate of 100 Hz.

The sample chamber sits below the process chamber. Samples are contained in a sample holder with either a metallic wire cage structure or a ceramic cup on top of a SiN pedestal.

Due to the relatively short µ*g* times, convective cooling with a He–Ar gas mixture is necessary to increase the cooling rate, aiming for solidification during the µ*g* phase.

### Oscillating drop method

The heater pulses during the nearly force-free cooling phase lead to an axial elongation of the sample, which leads predominantly to oscillations in the *Y*_2,0_ mode. Since in a force-free µ*g* environment all *Y*_2,m_ are degenerated,^14^ only one oscillation frequency *ν*_m _= *ν*_R_ (the Rayleigh frequency) can be observed. Under 1*g* EML, the sample is not force free and spherical, but deforms. This leads to a split and shift of the measured oscillation frequencies *v*_m_. A correction was developed^14^ and successfully proven by comparison of µ*g* with ground-based surface tension measurements in an EML device.^31^ Periodic sample movements within the positioning field lead to small, periodic forces on the sample. Under this condition, the application of the so-called Cummings and Blackburn correction^14^ results in a reduction of the surface tension values in the range of 2–3% when the measured surface oscillation frequency *v*_m_ instead of *ν*_R_ is used in the formula for the evaluation of Eq. 1. This correction was not applied to the data presented here. Also, the absence of excessive sample rotation is necessary for the validity of Eq. 1, which was the case in our experiments.^32^

Viscosity is evaluated from the damping time constant *τ* of the surface oscillations according to Eq. 2. There are, however, some subtleties and constraints in the application of Eqs. 1 and 2. For small enough viscosities, the viscosity does not change the Rayleigh frequency of the surface oscillations (τ^-1^ << *v*_R_).^33,34^

Evaluation of the viscosity from Eq. 2 requires the absence of turbulence. Moreover, derivation of this formula is based on small amplitude oscillations allowing to neglect the nonlinear term in the Navier–Stokes equation. In addition, a strictly Newtonian fluid is assumed for the viscosity analysis.^17^

### Reporting summary

Further information on experimental design is available in the Nature Research Reporting Summary linked to this article.

## Supplementary information

Reporting Summary

## Data Availability

The datasets generated during and analyzed during the current study are available from the corresponding author on reasonable request.

## Supplementary information

**Supplementary information** accompanies the paper on the *npj Microgravity* website (10.1038/s41526-019-0065-4).
